# Epidural analgesia supplemented with lidocaine reduces maternal fever during labor: a randomized controlled trial

**DOI:** 10.3389/fphar.2026.1839928

**Published:** 2026-06-22

**Authors:** Yufei Zhang, Di Yu, Bei Shen, Shuang Ma, Chunli Xu

**Affiliations:** 1 Department of Anesthesiology, Union Hospital, Tongji Medical College, Huazhong University of Science and Technology, Wuhan, China; 2 Department of Anesthesiology, The Third People’s Hospital of Hubei Province, Wuhan, China; 3 Department of Anesthesiology, Jiaxing Women and Children’s Hospital, Jiaxing, China; 4 Department of Obstetrics and Gynecology, Jiaxing Women and Children’s Hospital, Jiaxing, China

**Keywords:** epidural analgesia, intrapartum fever, labor, lidocaine, neonates

## Abstract

**Background:**

The co-administration of neuraxial drugs can enhance analgesic effects and reduce adverse events associated with epidural labor analgesia. This study aimed to explore the effects of epidural analgesia supplemented with lidocaine on maternal intrapartum fever.

**Methods:**

The control group received epidural analgesia with 0.1% ropivacaine and fentanyl 2 μg/mL, while Group L received epidural analgesia with 0.3% lidocaine, 0.1% ropivacaine, and fentanyl 2 μg/mL. Maternal temperature, pain intensity, fetal heart rate, and labor outcomes were recorded. Adverse events, if any, were also documented.

**Results:**

Initially, 400 women were enrolled in this study. Ultimately, 184 were assigned to the control group (n = 184), and 186 were assigned to Group L (n = 186). The incidence of intrapartum fever was lower in Group L than in the control group (3.8% vs. 9.2%; odds ratio = 0.384, 95% CI [0.155–0.950], *P* = 0.032). The onset of anesthesia was shorter in Group L than in the control group (9.8 ± 2.0 vs. 10.8 ± 2.3 min, *P* < 0.01). The visual analog scale scores 4 h after analgesia were lower in Group L than in the control group (*P* < 0.05).

**Conclusion:**

Epidural analgesia supplemented with lidocaine reduced the incidence of intrapartum fever and accelerated the onset of analgesia without increasing maternal or neonatal complications.

**Clinical Trial Registration:**

https://www.chictr.org.cn/, Identifier, ChiCTR2300075546.

## 1 Introduction

It is well known that maternal body temperature tends to rise during neuraxial labor analgesia. Elevated maternal temperature can increase oxygen consumption in both the mother and fetus, potentially leading to fetal distress and, in severe cases, neonatal intracranial hemorrhage or cerebral palsy (Chang et al., 2023). Currently, the specific mechanism of intrapartum fever remains unclear (Kristýna et al., 2021). It is generally believed that intrapartum fever is caused by sterile inflammation, characterized by elevated levels of proinflammatory cytokines (Zhou et al., 2019). Neuraxial analgesia is the most widely used method for pain relief (Krusic et al., 2026). The co-administration of neuraxial drugs can enhance analgesic effects and reduce adverse events associated with the block (Zhang et al., 2019). Previous studies (Li et al., 2021; Lange et al., 2017) have found that co-administration of neuraxial dexmedetomidine or magnesium sulfate reduces the incidence of maternal intrapartum fever. Additionally, one study indicated that neuraxial dexamethasone mitigated maternal temperature elevation during epidural labor analgesia (Wang et al., 2011).

Lidocaine is a classic local anesthetic that is widely used in clinical practice. Recent studies have shown that intravenous lidocaine plays a positive role in modulating the inflammatory response (Chu et al., 2020). An increasing number of studies have demonstrated that intravenous infusion of lidocaine exerts anti-inflammatory effects (Dunn and Durieux, 2017; Su et al., 2023). However, it is unclear whether epidural analgesia supplemented with lidocaine can decrease the incidence of intrapartum fever during labor analgesia. The aim of this study was to investigate the effects of epidural analgesia supplemented with lidocaine on intrapartum fever in women undergoing epidural labor analgesia.

## 2 Methods

The study was approved by the Ethics Board of Jiaxing Women and Children Hospital, and written informed consent was obtained from all participants. The trial was registered in the Chinese Clinical Trials Registry (Registration number: ChiCTR2300075546). From August 2023 to July 2024, a total of 400 full-term primiparous parturients demanding labor analgesia were enrolled in this study. Inclusion criteria were American Society of Anesthesiologists (ASA) physical status II, age 20–40 years, weight 55–100 kg and gestational age ≥37 weeks. Exclusion criteria included body temperature >37 °C, platelet count <80 × 10^9^/L, white blood cell count >12 × 10^9^/L, serious liver or renal dysfunction.

Randomization was carried out by opening an opaque, sealed envelope containing a sequential number. The allocation sequence was generated using an online randomization generator. Parturients, anesthesiologists, obstetricians, midwives and investigators were blinded to the group allocation. Study drugs were prepared by nurses, who were not involved in this study.

Entering the delivery room, non-invasive blood pressure, heart rate (HR), and pulse oxygen saturation (SpO_2_) were monitored at 30-min intervals, and peripheral venous access was established. When cervical dilation reached approximately 2 cm, epidural analgesia was administered at the L2–L3 interspace in the left lateral position using the loss-of-resistance to air technique. An epidural catheter was advanced 4 cm into the epidural space. A test dose of 3 mL of analgesic solution containing 100 µg of adrenaline was administered via the epidural catheter, followed by an initial loading dose of 10 mL of the analgesic solution. In Group L, the anesthetic solution consisted of 0.3% lidocaine, 0.1% ropivacaine, and 2 μg/mL fentanyl. In the control group, the anesthetic solution contained 0.1% ropivacaine and 2 μg/mL fentanyl. An electronic infusion pump was used to provide patient-controlled epidural analgesia to the parturients. The parameters of infusion pump were set as follows: an 8 mL/h background dose, a 6 mL bolus dose, and a 15-min lockout interval. If the visual analog scale (VAS) value 30 min after analgesia was >3, a bolus dose was administered. Maternal temperature was measured by infrared ear thermometer (Braun Inc., IRT6520, Germany) at 30-min intervals. The measurements were recorded immediately before analgesia (T0), 1 h after analgesia (T1), 2 h after analgesia (T2), 3 h after analgesia (T3), 4 h after analgesia (T4) and at 10 cm of cervical dilatation (T5). The onset of anesthesia was defined as the time from epidural bolus injection completion to VAS value <3. Hypotension was defined as a systolic blood pressure below 80% of baseline values and was treated with an intravenous dose of 40 µg phenylephrine. Supplemental oxygen was administered if maternal SpO_2_ fell below 94%. Pain intensity was assessed using the visual analog scale. Motor block was defined as a Bromage score (0 = No motor block; 1 = Unable to move the hip; 2 = Unable to move the hip and flex the knee; 3 = Unable to move the hip, flex the knee, and move the ankle) greater than one. The incidence of hypotension, respiratory depression, shivering, itching, nausea and vomiting was recorded. Apgar scores at 5 min were also recorded. Respiratory depression was defined as a respiratory rate <10 breaths/min and SpO_2_ < 92%. Intrapartum fever was defined as the maternal temperature from the ear canal of ≥38 °C, and white blood with a count <12 × 10^9^/L and plasma C-reactive protein less than 10 mg/L.

### 2.1 Sample size

The primary outcome was the incidence of intrapartum fever, and the secondary outcome was the analgesic effect. Based on our preliminary findings, which showed a decrease in the incidence of intrapartum fever from 12% to 4%, a sample size of 180 individuals per group was required to detect a statistically significant difference in the incidence of intrapartum fever with an alpha level of 0.05 and a power of 0.80. To accommodate for any dropouts, the sample size for each group was increased to 200.

### 2.2 Statistical analysis

Statistical analysis was performed using SPSS 20.0 (IBM, NY, USA) software. Normally distributed continuous variables were presented as mean and standard deviation (SD) and analyzed by *t*-test. Non-normally distributed continuous variables were presented as median [interquartile range] and analyzed by Mann-Whitney U test. Categorical data were presented as numbers and analyzed using Fisher’s exact test or Chi-square test. *P-*values <0.05 were considered statistically significant.

## 3 Results

**FIGURE 1:**
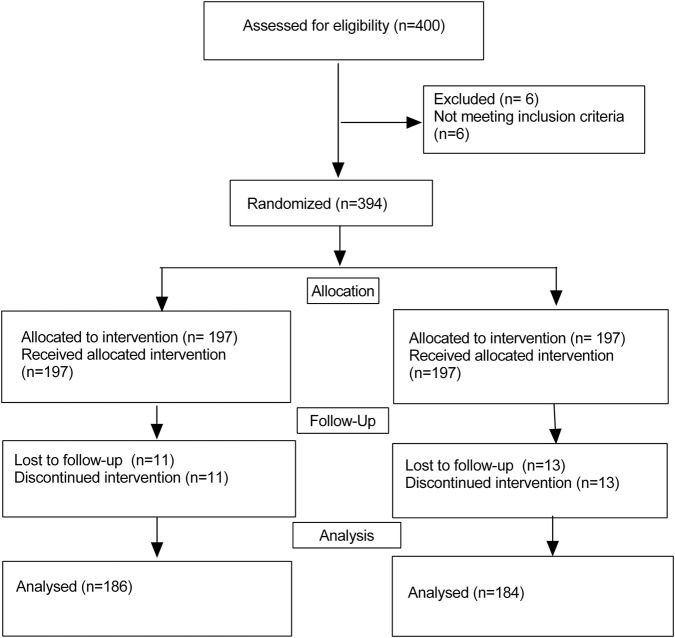
Flow diagram of study.

Initially, 400 women were enrolled, and 394 were randomly allocated in this study (Figure 1). A total of 24 women were lost to follow-up due to cesarean sections performed during labor. Ultimately, 370 women completed the study.

### 3.1 Characteristics of women

No statistically significant differences were observed between the two groups in age, weight, height, gestational weeks, stages of delivery, or Apgar scores (in Table 1). However, the onset of anesthesia was shorter in Group L than in the control group (9.8 ± 2.0 vs. 10.8 ± 2.3 min, *P* < 0.01), and the consumption of anesthetics was lower in Group L (74.8 ± 6.4 vs. 76.4 ± 6.7mL, *P* = 0.019).

**TABLE 1:** Data of women in both groups.

Variables	Group L (n = 186)	Control group (n = 184)	*P* Value
Age (year)	27.3 ± 3.4	27.4 ± 3.5	0.821
Height (cm)	160.3 ± 4.7	161.8 ± 4.8	0.141
Weight (kg)	68.5 ± 7.5	67.8 ± 7.6	0.754
Gestational age (week)	39.1 ± 0.9	39.2 ± 1.0	0.268
Onset of analgesia (min)	9.8 ± 2.0	10.8 ± 2.3	0.000*
First stage of labor (min)	389.7 ± 75.2	405.9 ± 77.7	0.058
Second stage of labor (min)	58.2 ± 15.3	60.5 ± 16.2	0.152
Third stage of labor (min)	13.7 ± 2.2	13.8 ± 2.1	0.659
Consumption of anesthetics (mL)	74.8 ± 6.4	76.4 ± 6.7	0.019*
5-min apgar scores	9.4 ± 0.7	9.3 ± 0.6	0.403

Data were presented as mean ± SD, or numbers, *P < 0.05.

### 3.2 Hemodynamics

**FIGURE 2:**
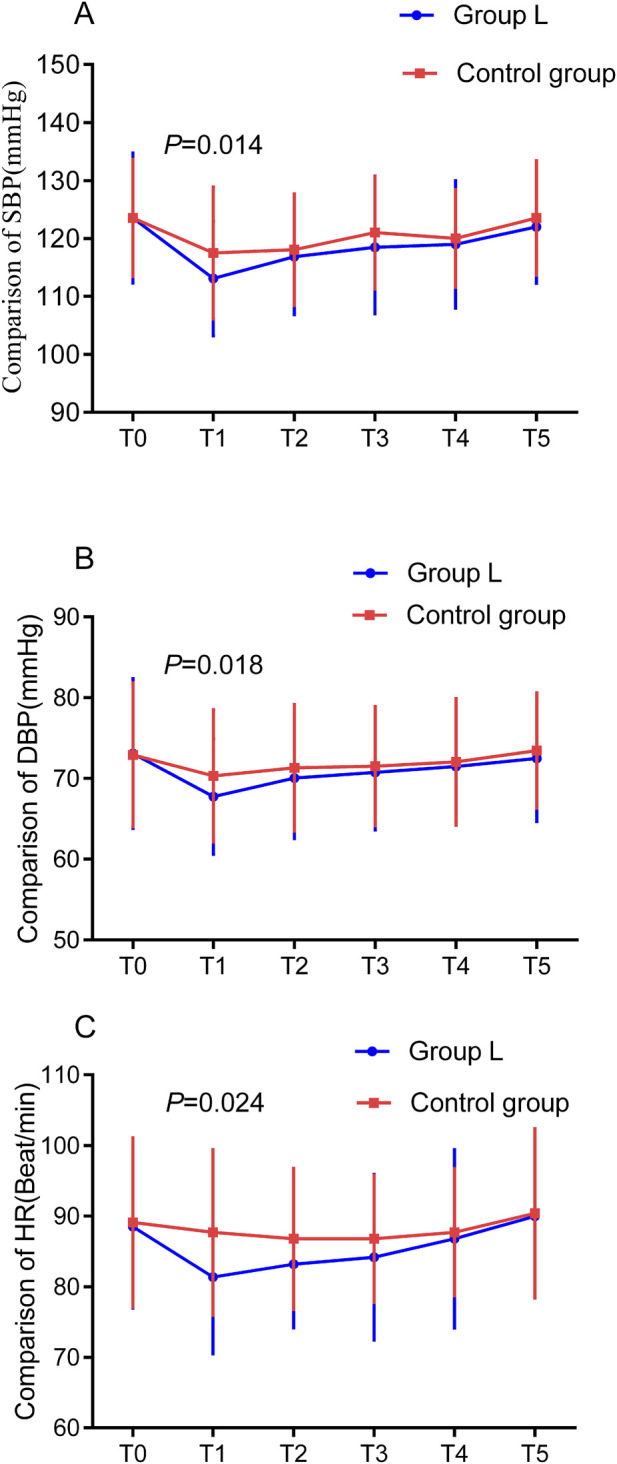
Comparison of Blood pressure and HR in both groups. Blood pressure and HR at T1 were lower in Group L than in the control group (*P < 0.05). While there were no statistically significant differences in blood pressure and HR at other time points between the two groups (*P* > 0.05). SBP: systolic blood pressure, DBP: diastolic blood pressure; HR: heart rate. T0: immediately before analgesia, T1: 1 h after analgesia, T2: 2 h after analgesia, T3: 3 h after analgesia, T4: 4 h after analgesia, T5: 10 cm of cervical dilatation.

At T1, blood pressure and HR were lower in Group L than in the control group (*P* < 0.05). No significant differences in blood pressure or HR were observed at other time points between the two groups (in Figure 2).

### 3.3 Analgesic effect

**FIGURE 3:**
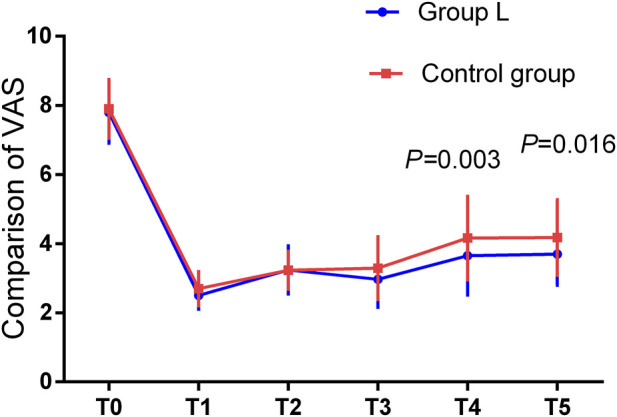
VAS at different time points. The VAS score was lower in Group L from T4 toT6 than in the control group (*P* < 0.05). T0: immediately before analgesia, T1: 1 h after analgesia, T2: 2 h after analgesia, T3: 3 h after analgesia, T4: 4 h after analgesia, T5: 10 cm of cervical dilatation.

The VAS scores from T4 toT5 were lower in Group L than in the control group (*P* < 0.05). There were no statistically significant differences in VAS scores at other time points between the two groups (in Figure 3).

### 3.4 Maternal temperature

**FIGURE 4:**
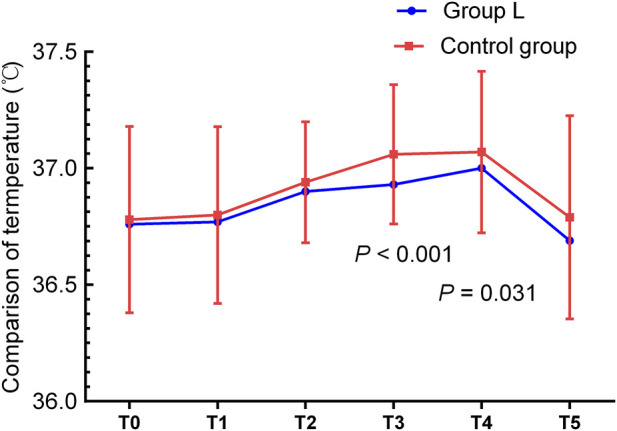
Maternal temperature at different time points. The temperature value was significantly lower at T4 in Group L than the control group (*P* < 0.05). There were no significant differences in maternal temperature at other time points between the two groups. T0: immediately before analgesia, T1: 1 h after analgesia, T2: 2 h after analgesia, T3: 3 h after analgesia, T4: 4 h after analgesia, T5: 10 cm of cervical dilatation.

At T3 and T4, temperature values were lower in Group L than in the control group (36.9 ± 0.3 vs. 37.1 °C ± 0.3 °C, *P* < 0.001; 37.0 ± 0.3 vs. 37.1 °C ± 0.3 °C, *P* = 0.031). Temperature values at other time points were similar in both groups, with no significant differences observed (*P* > 0.05) (in Figure 4).

### 3.5 Adverse events

Patients in group L showed a lower incidence of fever (3.8% vs. 9.2%, odds ratio = 0.384, 95% CI [0.155–0.950], *P* = 0.032). No significant differences were observed between the two groups regarding hypotension, itching, shivering, motor block, nausea, and vomiting (in Table 2).

**TABLE 2:** Adverse events of women and infants.

Index	Group L (n = 186)	Control group (n = 184)	*P -* value
Intrapartum fever (n)	7 (3.8%)	17 (9.2%)	0.032*
Vomiting and nausea (n)	11	9	0.655
Hypotension (n)	9	7	0.617
Itching (n)	6	5	0.766
Motor block (n)	1	0	0.999
Shivering (n)	2	4	0.449
Fetal destress (n)	1	2	0.623

Data are shown as number, *P < 0.05.

## 4 Discussion

This study showed that epidural analgesia supplemented with lidocaine reduced the incidence of intrapartum fever and accelerated the onset of analgesia without increasing maternal or neonatal complications.

The present study showed that the incidence of intrapartum fever was decreased in Group L. The mechanism of maternal intrapartum fever is not fully understood. The risk of maternal fever is increased in women receiving epidural analgesia compared to the not receiving analgesia; however, the association between epidural analgesia and maternal fever is complex and controversial (Zhao et al., 2022). The mechanism of intrapartum fever may be related to an imbalance of heat and cold following epidural analgesia. Firstly, after an epidural block, vasodilation caused an increase in heat loss. Secondly, patients’ anxiety and energy consumption were decreased by effective analgesia. On the other hand, pain enhanced the body’s stress response, which accelerated metabolism and produced more heat. The literature reported that maternal prenatal inflammatory biomarkers were associated with the risk of labor epidural-associated fever (Arce et al., 2019). TNF-α, IL-1β and IL-6 are pro-inflammatory cytokines that play a central role in intrapartum fever (Goetzl et al., 2010). Sultan and his colleagues proposed that anesthetic drug–induced metabolic dysfunction might trigger the release of inflammatory molecules, which was probably the cause of epidural-related maternal fever (Sultan et al., 2016). Wohlrab and his colleagues found that ropivacaine contributed to epidural-related maternal fever by activating multiple proapoptotic and inflammatory signaling pathways, whereas lidocaine suppressed the release of IL-6 and IL-8 (Wohlrab et al., 2020). One study suggests that lidocaine may modulate the inflammatory response and attenuate the release of inflammatory mediators by inhibiting inflammatory cells, modulating signaling pathways, and interfering with central neuroreceptor transmission (Wang et al., 2024). These studies implicate an anti-inflammatory effect of lidocaine. Li and his colleagues reported that dexmedetomidine decreased the incidence of intrapartum maternal fever (Li et al., 2021). Dexmedetomidine may inhibit the thermoregulatory center to reduce heat production by activating central α_2_-adrenoceptors and suppressing c-Jun N-terminal kinases (Zhang et al., 2014). A retrospective study shows that magnesium may play a protective role against the development of intrapartum fever (Lange et al., 2017). The mechanism by which magnesium sulfate regulates body temperature regulation is not fully known. One of mechanisms may be related to magnesium induced peripheral vasodilation. In addition, magnesium inhibits the systemic inflammatory response and reduces the febrile reaction in parturients (Lange et al., 2017). Although the incidence of intrapartum fever decreased by 5.4%, this reduction is clinically significant for lowering the rates of neonatal adverse events and cesarean sections.

The co-administration of neuraxial drugs can enhance analgesic effects and reduce adverse events associated with epidural block. In our study, VAS values 4 h after analgesia were significantly lower in Group L than in the control group. It indicated that lidocaine increased the analgesic effect of ropivacaine. Motor block occurred frequently when higher concentrations of local anesthetics were used for epidural analgesia. The current investigation found that adding lidocaine to epidural analgesia did not increase the risk of motor block. Additionally, the incidence of maternal hypotension, itching, shivering, nausea, and vomiting was not increased by epidural analgesia with lidocaine, consistent with earlier research (Cavusoglu et al., 2025; Gude et al., 2021; Zhang et al., 2025; Jiang et al., 2026; Ferreira Suárez et al., 2026). Neonatal outcomes did not differ between the two groups. This study indicated that epidural analgesia supplemented with lidocaine did not increase the incidence of adverse events in either mothers or neonates.

This study had several limitations. There was potential bias in measuring cervical dilation, which affected the timing of body temperature measurements and influenced the results. More neonatal assessments—such as neonatal intensive care unit admission, cord blood analysis, and neurodevelopmental evaluations—require further study. Additionally, levels of magnesium ion may influence the occurrence of intrapartum fever.

## 5 Conclusion

Epidural analgesia supplemented with lidocaine reduced the incidence of intrapartum fever and accelerated the onset of analgesia without increasing maternal or neonatal complications.

## Data Availability

The original contributions presented in the study are included in the article/supplementary material, further inquiries can be directed to the corresponding authors.

## References

[B1] Arce D. Y. Bellavia A. Cantonwine D. E. Napoli O. J. Meeker J. D. James-Todd T. (2019). Average and time-specific maternal prenatal inflammatory biomarkers and the risk of labor epidural associated fever. PLoS One 14 (11), e0222958. 10.1371/journal.pone.0222958 31689293 PMC6830771

[B2] Cavusoglu C. G. Arkan K. Bagli I. Erkmen A. D. Colak B. Andic O. (2025). The impact of epidural analgesia on the dynamics of labor and perinatal outcomes in nulliparous women: a prospective cohort study. Med. Kaunas. 61 (12), 2109. 10.3390/medicina61122109 PMC12734504 41470111

[B3] Chang X. Y. Wang L. Z. Xia F. Zhang Y. F. (2023). Factors associated with epidural-related maternal fever in low-risk term women: a systematic review. Int. J. Obstet. Anesth. 56, 103915. 10.1016/j.ijoa.2023.103915 37625990

[B4] Chu R. Umukoro N. Greer T. Roberts J. Adekoya P. Odonkor C. A. (2020). Intravenous lidocaine infusion for the management of early postoperative pain: a comprehensive review of controlled trials. Psychopharmacol. Bull. 50 (4 Suppl. 1), 216–259. 10.64719/pb.4391 33633427 PMC7901134

[B5] Dunn L. K. Durieux M. E. (2017). Perioperative use of intravenous lidocaine. Anesthesiology 126, 729–737. 10.1097/ALN.0000000000001527 28114177

[B6] Ferreira Suárez M. Guerra L. de Polsi F. Delgado R. Soust A. Solari M. (2026). Labor epidural analgesia and obstetric and neonatal outcomes in a public tertiary maternity in Uruguay: a retrospective cohort study (2020-2022). Int. J. Obstet. Anesth. 66, 104925. Published online. 10.1016/j.ijoa.2026.104925 41905131

[B7] Goetzl L. Manevich Y. Roedner C. Praktish A. Hebbar L. Townsend D. M. (2010). Maternal and fetal oxidative stress and intrapartum term fever. Am. J. Obstet. Gynecol. 202 (4), 363.e1–363.e3635. 10.1016/j.ajog.2010.01.034 20350644 PMC4594801

[B8] Gude P. Kaci C. S. B. Sieker M. Vogelsang H. Bellgardt M. Herzog-Niescery J. (2021). The influence of labor epidural analgesia on maternal, uteroplacental and fetoplacental hemodynamics in normotensive parturients: a prospective observational study. Int. J. Obstet. Anesth. 45, 83–89. 10.1016/j.ijoa.2020.10.011 33298344

[B9] Jiang X. Xiu Y. Jia C. Han B. Zu J. Zhang S. (2026). Analgesic efficacy and adverse effects of epidural ropivacaine combined with equipotent alfentanil or sufentanil for labor analgesia: a multicenter randomized controlled single-blind trial. Pain Ther. 15, 481–494. Published online. 10.1007/s40122-025-00812-9 41609980 PMC13009347

[B10] Kristýna H. Tereza B. Jan B. (2021). Epidural fever. Epidurální horečka. Ceska Gynekol. 86 (5), 355–361. 10.48095/cccg2021355 34736336

[B11] Krusic S. B. Milic J. Z. Sharpe E. E. Pejcic N. Z. Pujic B. P. Stojanovic-Tasic M. P. (2026). Association between the effectiveness of labor epidural analgesia and postpartum depression: a prospective cohort study. J. Anesth. 40, 94–105. 10.1007/s00540-025-03563-1 40782149

[B12] Lange E. M. S. Segal S. Pancaro C. Wong C. A. Grobman W. A. Russell G. B. (2017). Association between intrapartum magnesium administration and the incidence of maternal fever: a retrospective cross-sectional study. Anesthesiology 127 (6), 942–952. 10.1097/ALN.0000000000001872 28863031

[B13] Li L. Yang Z. Zhang W. (2021). Epidural dexmedetomidine for prevention of intrapartum fever during labor analgesia: a randomized controlled trial. Pain Ther. 10 (1), 391–400. 10.1007/s40122-020-00215-y 33188493 PMC8119513

[B14] Su K. Li X. T. Hong F. X. Jin M. Xue F. S. (2023). Lidocaine pretreatment attenuates inflammatory response and protects against sepsis-induced acute lung injury via inhibiting potassium efflux-dependent NLRP3 activation. Inflamm. Res. 72, 2221–2235. 10.1007/s00011-023-01810-3 37930383

[B15] Sultan P. David A. L. Fernando R. Ackland G. L. (2016). Inflammation and epidural-related maternal fever: proposed mechanisms. Anesth. Analg. 122 (5), 1546–1553. 10.1213/ANE.0000000000001195 27101499

[B16] Wang L. Z. Hu X. X. Liu X. Qian P. Ge J. M. Tang B. L. (2011). Influence of epidural dexamethasone on maternal temperature and serum cytokine concentration after labor epidural analgesia. Int. J. Gynaecol. Obstet. 113, 40–43. 10.1016/j.ijgo.2010.10.026 21306709

[B17] Wang J. Bian Q. Chen X. Feng Y. Zhang L. Chen P. (2024). The mechanism of perioperative intravenous lidocaine in regulating the inflammatory response: a review. Med. Baltim. 103 (36), e39574. 10.1097/md.0000000000039574 39252226 PMC11384871

[B18] Wohlrab P. Boehme S. Kaun C. Wojta J. Spittler A. Saleh L. (2020). Ropivacaine activates multiple proapoptotic and inflammatory signaling pathways that might subsume to trigger epidural-related maternal fever. Anesth. Analg. 130 (2), 321–331. 10.1213/ANE.0000000000004402 31498191

[B19] Zhang X. Wang J. Qian W. Zhao J. Sun L. Qian Y. (2014). Dexmedetomidine inhibits tumor necrosis factor-alpha and interleukin 6 in lipopolysaccharide-stimulated astrocytes by suppression of c-Jun N-terminal kinases. Inflammation 37 (3), 942–949. 10.1007/s10753-014-9814-4 24429914

[B20] Zhang T. Yu Y. Zhang W. Zhu J. (2019). Comparison of dexmedetomidine and sufentanil as adjuvants to local anesthetic for epidural labor analgesia: a randomized controlled trial. Drug Des. Dev. Ther. 13, 1171–1175. 10.2147/DDDT.S197431 31043770 PMC6469486

[B21] Zhang Y. Y. Cheng X. L. Liu L. Li L. Diao Y. (2025). Esketamine as an opioid-sparing adjuvant for epidural labour analgesia: a randomised, double-blind trial evaluating postpartum depression. BMC Anesthesiol. 26 (1), 73. 10.1186/s12871-025-03590-1 41462089 PMC12859885

[B22] Zhao B. Li B. Wang Q. Song X. (2022). The relationship between epidural analgesia and intrapartum maternal fever and the consequences for maternal and neonatal outcomes: a prospective observational study. J. Matern. Fetal Neonatal Med. 35 (25), 5354–5362. 10.1080/14767058.2021.1879042 33504250

[B23] Zhou X. Li J. Deng S. Xu Z. Liu Z. (2019). Ropivacaine at different concentrations on intrapartum fever, IL-6 and TNF-α in parturient with epidural labor analgesia. Exp. Ther. Med. 17, 1631–1636. 10.3892/etm.2018.7121 30783430 PMC6364190

